# Uncultivated Viral Populations Dominate Estuarine Viromes on the Spatiotemporal Scale

**DOI:** 10.1128/mSystems.01020-20

**Published:** 2021-03-16

**Authors:** Mengqi Sun, Yuanchao Zhan, David Marsan, David Páez-Espino, Lanlan Cai, Feng Chen

**Affiliations:** a Institute of Marine and Environmental Technology, University of Maryland Center for Environmental Science, Baltimore, Maryland, USA; b Department of Energy, Joint Genome Institute, Berkeley, California, USA; c Mammoth Biosciences, Inc., South San Francisco, California, USA; d State Key Laboratory of Marine Environmental Science, Institute of Marine Microbes and Ecospheres, Xiamen University (Xiang’an), Xiamen, China; University of California, Davis

**Keywords:** virome, virioplankton, Chesapeake Bay, Delaware Bay, estuarine ecosystem, viral community, metagenomics, microbial ecology, high-throughput sequencing, viral populations, estuarine

## Abstract

Viruses are ubiquitous and abundant in the oceans, and viral metagenomes (viromes) have been investigated extensively via several large-scale ocean sequencing projects. However, there have not been any systematic viromic studies in estuaries. Here, we investigated the viromes of the Delaware Bay and Chesapeake Bay, two Mid-Atlantic estuaries. Deep sequencing generated a total of 48,190 assembled viral sequences (>5 kb) and 26,487 viral populations (9,204 virus clusters and 17,845 singletons), including 319 circular viral contigs between 7.5 kb and 161.8 kb. Unknown viruses represented the vast majority of the dominant populations, while the composition of known viruses, such as pelagiphage and cyanophage, appeared to be relatively consistent across a wide range of salinity gradients and in different seasons. A difference between estuarine and ocean viromes was reflected by the proportions of *Myoviridae*, *Podoviridae*, *Siphoviridae*, *Phycodnaviridae*, and a few well-studied virus representatives. The difference in viral community between the Delaware Bay and Chesapeake Bay is significantly more pronounced than the difference caused by temperature or salinity, indicating strong local profiles caused by the unique ecology of each estuary. Interestingly, a viral contig similar to phages infecting Acinetobacter baumannii (“Iraqibacter”) was found to be highly abundant in the Delaware Bay but not in the Chesapeake Bay, the source of which is yet to be identified. Highly abundant viruses in both estuaries have close hits to viral sequences derived from the marine single-cell genomes or long-read single-molecule sequencing, suggesting that important viruses are still waiting to be discovered in the estuarine environment.

**IMPORTANCE** This is the first systematic study about spatial and temporal variation of virioplankton communities in estuaries using deep metagenomics sequencing. It is among the highest-quality viromic data sets to date, showing remarkably consistent sequencing depth and quality across samples. Our results indicate that there exists a large pool of abundant and diverse viruses in estuaries that have not yet been cultivated, their genomes only available thanks to single-cell genomics or single-molecule sequencing, demonstrating the importance of these methods for viral discovery. The spatiotemporal pattern of these abundant uncultivated viruses is more variable than that of cultured viruses. Despite strong environmental gradients, season and location had surprisingly little impact on the viral community within an estuary, but we saw a significant distinction between the two estuaries and also between estuarine and open ocean viromes.

## INTRODUCTION

Estuaries are vital links between marine and terrestrial ecosystems and are among the most productive ecosystems on the planet (1). Estuarine systems encompass a complex spectrum of environmental gradients, creating distinct microbial habitats, and the frequent fluctuation of environmental conditions causes unique selective pressures to be exerted on organisms (2). In a highly dynamic estuarine environment, changes in environmental factors can trigger genetic and ecological shifts in microbial communities (3). Compared to those in coastal marine and river waters, bacterial densities and growth rates are generally higher in estuaries and tend to be highest in surface waters and turbid regions (4). The bacterioplankton community in the Chesapeake estuary exhibits a strong and repeatable seasonal pattern but less variation across the spatial scale (5, 6). Virioplankton are usually 1 order of magnitude more abundant than bacterioplankton (7). The abundance of virioplankton in the Chesapeake Bay is in the range of 10^6^ to 10^8^ virus-like particles (VLPs) per milliliter (8, 9), which can be 10 to 1,000 times more abundant than the viral concentration in the open ocean (7). Virioplankton are an active and dynamic component of estuarine microbiomes and are responsive to changes in environmental factors and the bacterial community (10–12). They are an important part of the trophic system in estuaries, as they are responsible for bacterial mortality at a level similar to that of protist grazing (9, 13).

The Chesapeake Bay has a rich history of pioneer studies in virioplankton ecology. Efforts to understand the diversity of the virioplankton community in estuarine environments can be traced back 20 years ago, when Wommack et al. first applied pulsed-field gel electrophoresis (PFGE) to analyze how the Chesapeake Bay virioplankton community changed with time and location (11). While PFGE provides only viral community fingerprints based on the separation of viral genome sizes, changes in viral populations over time and space have been observed (11). In a later study, randomly amplified polymorphic DNA (RAPD) PCR was applied to investigate the dynamics of virioplankton communities in the Chesapeake Bay (14). It was found that the virioplankton community in the Bay exhibited stronger temporal variations than spatial variations (14), a pattern similar to the spatiotemporal variations seen for the Chesapeake Bay bacterioplankton community (6). The first metagenomics study on estuarine virioplankton was conducted in the Chesapeake Bay by sequence analysis of one sample pooled from nine different locations of the Bay (10). Despite the limitation of low sequencing coverage in the early days of viromic study, it was found that the Chesapeake Bay virome contains a high proportion of unknown and novel sequences. Among the viral sequences, more than 90% were found to be most similar to tailed phage from the *Caudovirales* order (10). Compared to the virioplankton community of the Chesapeake Bay, not much is known about that of the Delaware Bay.

In the past 10 years, the development of new sequencing technologies has greatly advanced our understanding of microbial diversity in nature. Using next-generation sequencing (NGS) technologies, a number of large-scale ocean sequencing projects (e.g., Global Ocean Sampling Expedition, Malaspina Expedition, Pacific Ocean Virome, and Tara Ocean’s Global Ocean Virome [GOV]) have made viral metagenomic databases increasingly accessible, revealing important findings about the diversity and the spatial and temporal distribution of ocean viruses (15–19). The most recent study, Tara Ocean’s GOV 2.0, shows that marine viral communities can be separated into five ecological zones, although no estuarine samples were included (19). Meanwhile, many viromic studies have shown that the most abundant viral species in the ocean still remain unknown (16, 20). Large-scale sequencing efforts generally include only a few sampling sites at coastal and brackish locations (10, 15, 21–25), but there has not been any systematic study of spatial and temporal variation of virus communities in dynamic estuarine environments using deep-sequencing technology (Table 1). In this study, we investigated the diversity and spatiotemporal variation of virioplankton communities in two temperate estuaries, Delaware Bay and Chesapeake Bay, using next-generation sequencing technology.

**TABLE 1:** Summary of estuarine metagenomic viral data sets to date^*a*^

Publication (reference)	Sample site(s)	Salinity (ppt)	Study type	Sequencing method
Bench et al., 2007 (10)	Chesapeake Bay (9 stations combined)	NA	Environmental	Sanger
Williamson et al., 2008 (15) (GOS)	Bay of Fundy, Canada	NA	Environmental	Sanger
	Delaware Bay	NA		
	Chesapeake Bay	3.47		
McDaniel et al., 2008 (24)	Tampa Bay	NA	Induced virome	454 GS20
Cai et al., 2016 (21)	Jiulong Estuary, China	25.50	Environmental	454 GS FLX
Hwang et al., 2016 (22)	Goseong Bay, Korea (6 stations combined)	34	Environmental	Illumina HiSeq 2000
Zeigler Allen et al., 2017 (25) (BSV)	Baltic Sea (10 separate stations)	0–34.35 (10 samples)	Environmental	454 GS FLX
This study (DEV)	Delaware Bay (10 separate stations); Chesapeake Bay (6 separate stations)	0.2–30.4 (16 samples)	Environmental	Illumina HiSeq 2500

a Abbreviations: GOS, Global Ocean Sampling; BSV, Baltic Sea Virome; DEV, Delmarva Estuarine Virome; NA, not available.

The Delaware Bay and the Chesapeake Bay are separated by the Delmarva Peninsula, and they differ in many aspects. As the second largest estuary on the U.S. Atlantic coast, the Delaware Bay is an archetypal, funnel-shaped, well-mixed coastal plain estuary (26). It is heavily urbanized at the upper bay, yet it supports important wetlands and fisheries in the lower bay, and its drainage basin is dominated by agricultural activity (27). The Delaware River, the main river input to the Delaware Bay, is among the worst-polluted waterways in the nation due to the release of toxic chemicals from the surrounding industries (28). The Chesapeake Bay is the largest and most productive estuary in the United States, featuring shallow waters with a mean depth of 6.5 m. It is a partially mixed estuary featuring dynamic patterns of internal transport and a long (∼180-day) water residence time (29, 30). Annual freshwater flow from the Susquehanna River is highly variable, impacting the ecology of the bay (31). The Chesapeake Bay watershed is about 80 times larger than the Delaware Bay (32). A large portion of the Chesapeake Bay is nutrient limited, while the Delaware Bay has higher nutrient and turbidity levels (33). It is unknown how these profound abiotic differences in the two different estuarine ecosystems impact the virioplankton communities.

In this study, 16 virioplankton samples were collected from the Delaware Bay and the Chesapeake Bay from low-, medium-, and high-salinity sites during three different seasons. High-throughput sequencing with deep-sequencing coverage of these estuarine samples enabled us to analyze the spatiotemporal variation of the viral community in the two large estuarine ecosystems.

## RESULTS

### Overview of sampling conditions and microbial counts.

**FIG 1:**
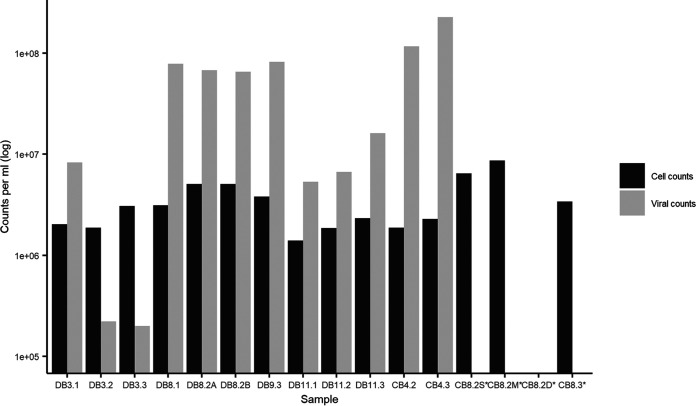
Bacterial and viral and count data in Delaware Bay (DB) and Chesapeake Bay (CB) determined by flow cytometer. Cells per milliliter and viral particles per milliliter are plotted on a logarithmic scale. Asterisks indicate that cell counts for CB8.2D and viral counts for CB8.2S-D and CB8.3 are missing.

Sixteen virioplankton community samples were collected from the Delaware and Chesapeake bays under a wide range of environmental conditions, with temperatures ranging from 4.0°C to 27.3°C and salinity ranging from 0.2 to 30.0 ppt (Table 2). Bacterial cell counts ranged from 1.4 × 10^6^ to 8.7 × 10^6^ cells per ml, while viral counts ranged from 1.9 × 10^5^ to 2.3 × 10^8^ per ml, showing a much wider variance than bacterial counts. As expected, the viral concentration is lower in winter months than in warmer seasons and is approximately 15-fold higher (ranging from 0.07 to 99.13; average, 21.10) than the bacterial concentration (Fig. 1). In the Delaware Bay, viral and bacterial abundances remained consistent during the summer and increased with the salinity gradient during the winter. In the Chesapeake Bay, samples from three different sampling depths were taken at station 8.2 in August, and stratification in the water column can be seen from the salinity data (Table 2). The surface low-salinity water contained higher concentrations of nitrate and chlorophyll *a* and a higher bacterial count than the middle (13.3-m) and deep (22.5-m) water (see Table S1 in the supplemental material). Fewer surface samples (*n* = 4) were taken from the Chesapeake Bay than the Delaware Bay (*n* = 10). No November samples were taken from the Chesapeake Bay.

**TABLE 2:** Sample site information and sequencing results

Sample	Yr	Date	Temp (°C)	Salinity (ppt)	No. of reads (millions)	% of low-quality reads (*Q* < 12)	No. of scaffolds (thousands)	Scaffold total size (Mb)
DB3.1	2014	19 Mar	4.4	0.2	135	1.20	954	652
DB3.2	2014	21 Mar	4.0	20.0	150	1.20	1,276	903
DB3.3	2014	22 Mar	4.0	30.4	146	1.30	899	595
DB8.1	2014	28 Aug	25.3	0.2	124	1	965	689
DB8.2A	2014	30 Aug	24.3	21.5	120	1.20	937	720
DB8.2B	2014	31 Aug	24.5	22.0	140	1.30	974	659
DB9.3	2014	1 Sep	24.3	28.8	210	1.80	1,365	964
DB11.1	2014	1 Nov	15.1	0.3	131	1.10	827	590
DB11.2	2014	2 Nov	13.8	15.4	218	1.50	1,816	1,267
DB11.3	2014	3 Nov	13.5	30.0	135	1	1,150	808
CB4.2	2015	12 Apr	8.5	9.1	59	1	658	509
CB4.3	2015	15 Apr	10.8	25.4	64	0.60	573	395
CB8.2S	2015	19 Aug	27.3	10.4	66	0.80	688	537
CB8.2M	2015	19 Aug	26.3	15.5	68	0.60	764	581
CB8.2D	2015	19 Aug	26.3	18.1	87	1.20	866	633
CB8.3	2015	22 Aug	26.6	26.7	62	0.70	690	536

10.1128/mSystems.01020-20.5 TABLE S1 Environmental conditions of DEV samples. Detailed information can be found at http://dmoserv3.bco-dmo.org/jg/serv/BCO-DMO/Coast_Bact_Growth/newACT_cruises_rs.html0%7Bdir=dmoserv3.whoi.edu/jg/dir/BCO-DMO/Coast_Bact_Growth/,info=dmoserv3.bco-dmo.org/jg/info/BCO-DMO/Coast_Bact_Growth/new_ACT_cruises%7D. Download Table S1, XLSX file, 0.01 MB. Copyright © 2021 Sun et al. 2021 Sun et al. https://creativecommons.org/licenses/by/4.0/ This content is distributed under the terms of the Creative Commons Attribution 4.0 International license.

### Sequencing results and viral contig identification.

Illumina HiSeq sequencing of the 16 viral samples produced 1,924 billion reads (150 bp, paired end) in total, which was named the Delmarva Estuarine Virome (DEV). The Delaware Bay samples yielded over twice as much sequencing depth as the Chesapeake Bay samples, with an average of 151 million reads for the Delaware Bay and an average of 68 million reads for the Chesapeake Bay. An average of 690 Mbp worth of contigs were assembled per sample. An overview of sequencing and assembly results is shown in Table 2.

An average of 3,012 viral contigs were identified for each sample using the approach described in the IMG/VR database (Table S3) (34, 35). Rarefaction curves showed that the sampling of DEV is close to saturation (Fig. S2).

10.1128/mSystems.01020-20.7 TABLE S3 Number of viral clusters and singletons and percentage of trimmed reads that map to viral populations. Download Table S3, DOCX file, 0.01 MB. Copyright © 2021 Sun et al. 2021 Sun et al. https://creativecommons.org/licenses/by/4.0/ This content is distributed under the terms of the Creative Commons Attribution 4.0 International license.

10.1128/mSystems.01020-20.2 FIG S2 Rarefaction curves of each sample. Rarefaction curves were produced using data from the M5NR database, representing species data of taxonomic categories from 16 viral metagenomes. The cutoffs used were as follows: alignment length, 15 bp; E value, E−5; percent identity, 60%. Download FIG S2, JPG file, 0.2 MB. Copyright © 2021 Sun et al. 2021 Sun et al. https://creativecommons.org/licenses/by/4.0/ This content is distributed under the terms of the Creative Commons Attribution 4.0 International license.

### Viral cluster network.

**FIG 2:**
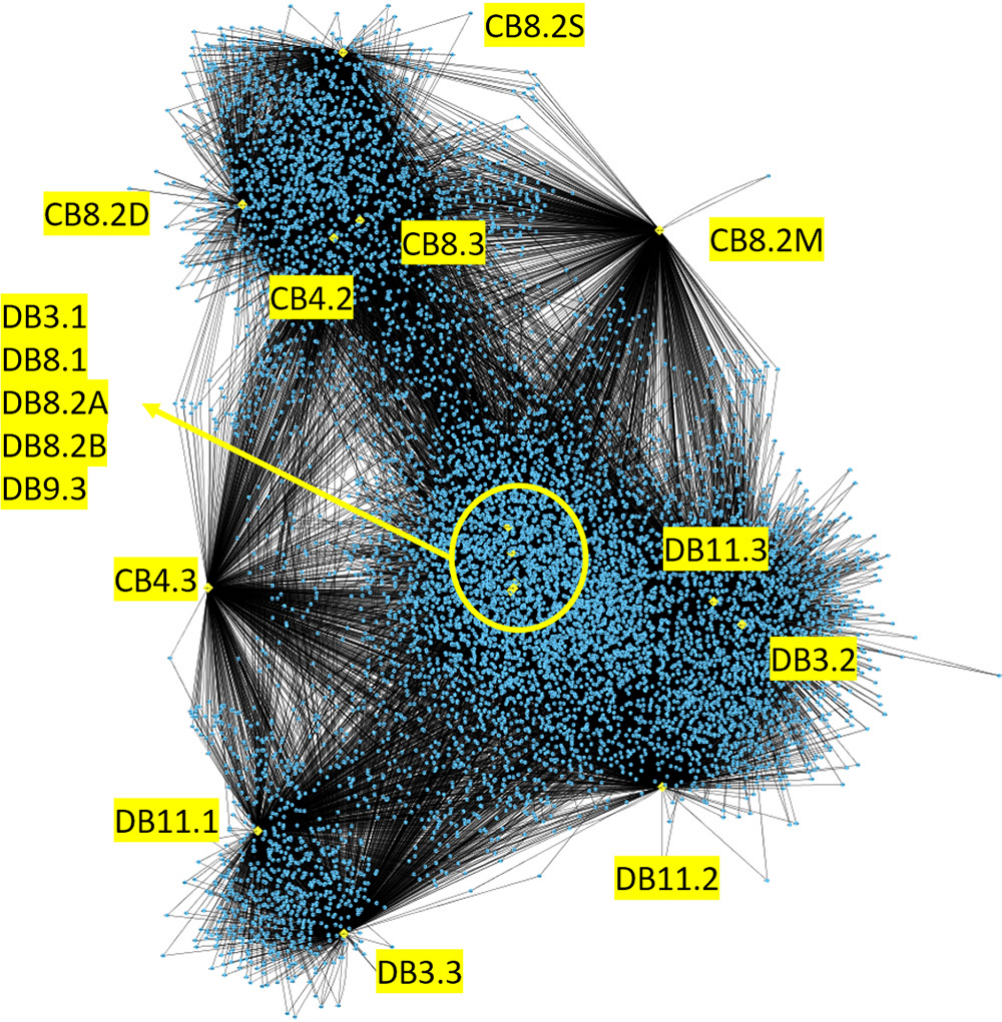
Cluster network of viral clusters and samples visualized using Cytoscape. Yellow nodes represent sampling stations; blue nodes represent viral clusters; edges (black lines connecting the nodes) represent their association. Singletons were omitted from the visualization for clarity.

To explore the diversity of contigs recovered from the DEV samples, we classified viral contigs into clusters and singletons based on sequence similarity (see Materials and Methods). A cluster is a group of DEV contigs (at least two contigs) that share high sequence similarity, while a singleton is a contig that does not belong to a cluster. From the 48,190 viral contigs (16 samples combined), 9,204 viral clusters and 17,845 singletons were detected. The number of clusters for each sample ranged from 697 to 2,960, while the number of singletons ranged from 419 to 3,115, reflecting a large number of viral contigs that are unique to their sample (Table S3). Sample DB11.2 produced the largest number of viral contigs (2,106) and also the largest number of singletons (3,115), suggesting the presence of a rich mid-bay viral diversity not found elsewhere (Table S3). It should be noted that since the viral contigs are assembled from short reads (150 bp), there is a limited amount of complete or nearly complete viral genomes, so it is likely that the numbers of singletons are overestimated when different portions of the same viral genome are not clustered together. A bipartite network was used to visualize the association between samples and clusters (Fig. 2). Delaware Bay summer samples seem to share many of the clusters with each other. Chesapeake Bay samples cluster distinctly from Delaware Bay samples and appear to show less similarity to each other than the Delaware Bay samples do. Strangely, the two samples DB3.3 and DB11.1 were grouped together and away from the other samples, despite having little in common (Fig. 2).

### Viral populations.

By combining the viral cluster and singleton information, a total of 26,487 viral populations were identified in the DEV samples (Table S4). An average of 26.2% trimmed reads mapped to viral populations in each sample (Table S3), indicating that nearly three-quarters of sequencing reads were not identified as viral at the current setting. Among the viral populations, 319 circular viral genomes were predicted via sequence overlaps. The length of circular viral genomes ranged from 7.5 kb to 161.8 kb, and they were mostly present in low abundance (average fragments per kilobase per million [FPKM], ≤20), with one exception (Ga0070751_1000196).

10.1128/mSystems.01020-20.8 TABLE S4 Length distribution of viral populations. Download Table S4, DOCX file, 0.01 MB. Copyright © 2021 Sun et al. 2021 Sun et al. https://creativecommons.org/licenses/by/4.0/ This content is distributed under the terms of the Creative Commons Attribution 4.0 International license.

A BLASTN search of population Ga0070751_1000196 against the NCBI-nr database showed the closest hit to podovirus Acinetobacter baumannii phage vB_AbaP_Acibel007, with a query cover of 47%, while the top 50 hits were various other Acinetobacter phages. Annotation by RAST showed that this genome had a total of 52 open reading frames (ORFs), of which only 8 proteins are known (36) (Fig. S3). Its host could not be predicted by the IMG/VR method (35). A search against the Tara Ocean Virome (TOV) and IMG/VR databases returned no results other than hits to its own sequence. The presence of a uniquely present, novel, and abundant viral population in the Delaware Bay is intriguing and remains to be explored.

10.1128/mSystems.01020-20.3 FIG S3 Whole genome of putative Acinetobacter (“Iraqibacter”) phage (accession number Ga0070751_1000196). Middle circle is GC content, inner circle is GC skew. Download FIG S3, TIF file, 0.5 MB. Copyright © 2021 Sun et al. 2021 Sun et al. https://creativecommons.org/licenses/by/4.0/ This content is distributed under the terms of the Creative Commons Attribution 4.0 International license.

### Spatiotemporal distribution of abundant viral populations.

**FIG 3:**
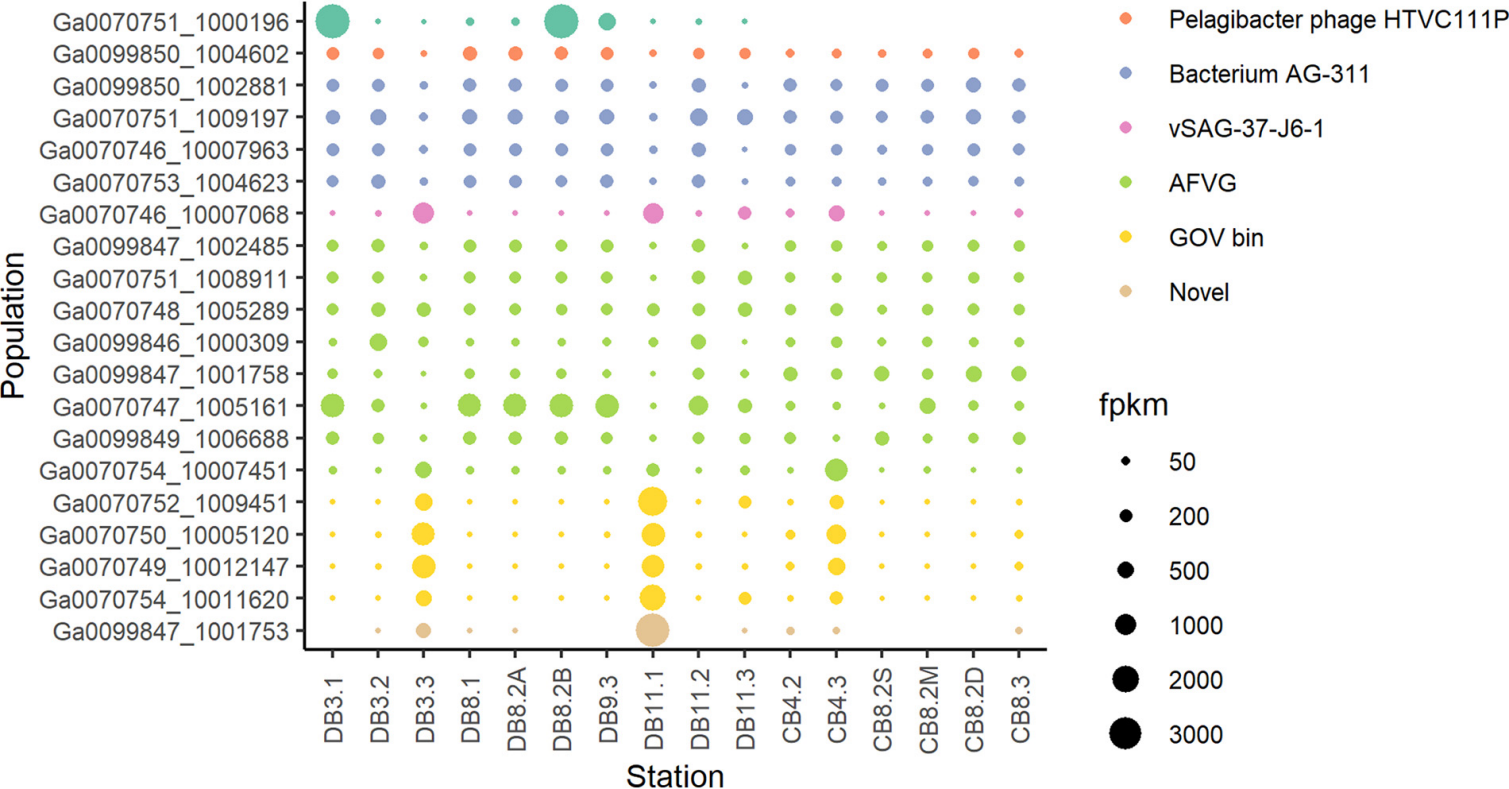
Relative abundance bubble plot of the top 20 most abundant viral populations for all 16 samples. The sizes of the bubbles correspond to the FPKM (fragments per kilobase million) for each sample, and colors correspond to the top BLAST hit of said viral population.

The relative distribution frequencies of the top 20 most abundant viral populations in these 16 estuarine samples were compared (Fig. 3). In the Delaware Bay, abundance variation in summer samples appears to be more consistent across the salinity gradient than that of spring or fall samples (Fig. 3). The relative abundances of these top 20 viral populations seem to be more variable in the Delaware Bay than in the Chesapeake Bay (Fig. 3).

When identification of the most abundant viruses was attempted by BLASTN search against the NCBI-nr database, these viruses were mostly found to share the closest similarity to other viral metagenomic sequences or to prokaryotes discovered using non-culture-based methods such as single-cell genomics and single-molecule sequencing (Table 3). Of the top 20 abundant virus populations, 4 shared the closest similarity to bacterium AG-311-K16, a marine cyanobacterium isolated using single-cell technology (37), 1 shared the closest similarity with vSAG 37-J6, a virus discovered using single-virus genomics (38), 8 matched viral sequences derived from assembly-free single-molecule sequencing (39), 4 matched uncultured viral populations from GOV (19), and 1 was completely novel. The only two readily identifiable cultured virus isolates in the top 20 were a putative Acinetobacter phage (Ga0070751_1000196) and *Pelagibacter* phage HTVC111P (Ga0099850_1004602). The putative Acinetobacter phage was found to be highly abundant in several Delaware Bay samples (the most abundant population in samples DB3.1 and DB8.2B) but was not present in Chesapeake Bay samples. In addition, a diel variation was noticed in samples DB8.2A and DB8.2B.

**TABLE 3:** Nucleotide BLAST results of top 20 abundant viral populations against NCBI-nr database

Viral population	Length (bp)	Total FPKM^*a*^	Top hit	Query cover (%)	E value	% identity
Ga0070747_1005161	5,953	9,474	Marine virus AFVG_25M393	4	3.00E−35	75
Ga0070751_1000196	42,033	7,894	Acinetobacter phage vB_AbaP_Acibel007	47	0.00E+00	73
Ga0070751_1009197	5,120	4,862	Bacterium AG-311-K16 Ga0172223_11	90	0.00E+00	80
Ga0099847_1001753	7,593	3,814	None			
Ga0099850_1002881	8,091	3,508	Bacterium AG-311-K16 Ga0172223_11	90	0.00E+00	77
Ga0070750_10005120	7,119	3,497	Prokaryotic dsDNA virus sp. isolate GOV_bin_15	54	0.00E+00	73
Ga0070752_1009451	5,331	3,343	Prokaryotic dsDNA virus sp. isolate Tp1_138_SUR_25606_1	65	6.00E−164	71
Ga0070749_10012147	5,544	3,042	Prokaryotic dsDNA virus sp. isolate GOV_bin_3107	3	3.00E−29	76
Ga0070748_1005289	5,790	2,875	Marine virus AFVG_117M37	86	0.00E+00	77
Ga0070746_10007963	6,108	2,797	Bacterium AG-311-K16 Ga0172223_11	58	0.00E+00	80
Ga0070754_10011620	5,489	2,618	Prokaryotic dsDNA virus sp. isolate GOV_bin_2950	39	3.00E−123	70
Ga0099847_1001758	7,589	2,580	Marine virus AFVG_117M42	97	0.00E+00	75
Ga0099847_1002485	6,383	2,551	Marine virus AFVG_117M61	39	0.00E+00	74
Ga0099849_1006688	5,235	2,485	Marine virus AFVG_25M322	100	0.00E+00	80
Ga0070746_10007068	6,491	2,343	Uncultured virus clone vSAG-37-J6-1	57	0.00E+00	70
Ga0070753_1004623	6,993	2,269	Bacterium AG-311-K16 Ga0172223_13	39	0.00E+00	80
Ga0070751_1008911	5,219	2,166	Marine virus AFVG_117M42	56	0.00E+00	78
Ga0099846_1000309	20,226	2,129	Marine virus AFVG_25M87	43	0.00E+00	83
Ga0099850_1004602	6,449	2,127	Pelagibacter phage HTVC111P	86	0.00E+00	78
Ga0070754_10007451	7,156	2,077	Marine virus AFVG_25M13	52	0.00E+00	71

a FPKM, fragments per kilobase million.

**FIG 4:**
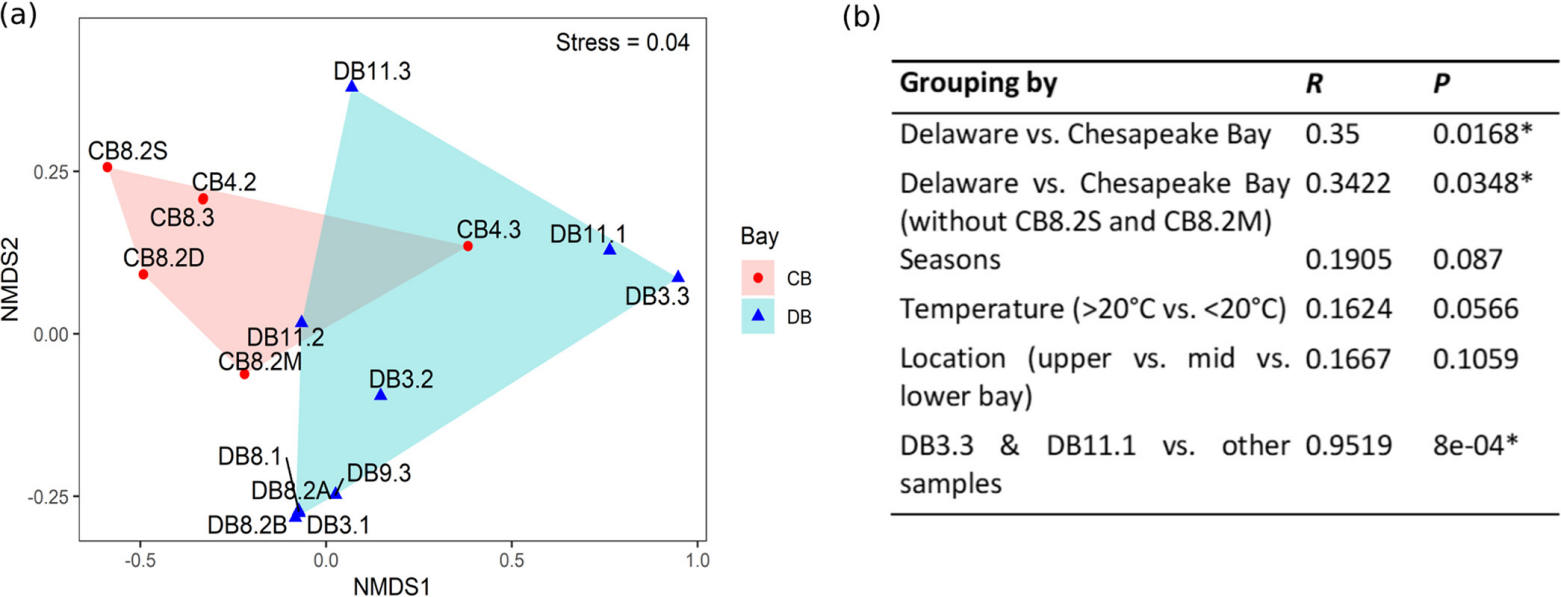
(a) Nonmetric multidimensional scaling (NMDS) plot made from top 5,000 most abundant viral populations. Stress level is indicated. DB, Delaware Bay; CB, Chesapeake Bay. Convex hulls are plotted around samples of each bay. (b) Analysis of similarity (ANOSIM) test based on top 5,000 most abundant viral populations (*, *P < *0.05).

Based on the top 5,000 most abundant populations, the 16 viromes clustered according to their bay of origin (Fig. 4a). Delaware Bay summer samples clustered together, but otherwise, samples generally did not cluster according to season or salinity (Fig. 4a). This was further confirmed by an analysis of similarity (ANOSIM) test; dissimilarity between groups was significant only when grouping samples by bay of origin (Fig. 4b). Inexplicably, samples DB3.1 and DB11.1 clustered together and away from other samples, the two of them showing significant dissimilarity with other samples (Fig. 4a and b).

Redundancy analysis (RDA) revealed the putative Acinetobacter phage (Ga0070751_1000196) and the most abundant viral population (Ga0070747_1005161) to be outliers with regard to their relationship with environmental parameters (Fig. S4). Their variance is not significantly (*P < *0.05) correlated with chlorophyll *a* concentrations, despite what the RDA figure may suggest.

10.1128/mSystems.01020-20.4 FIG S4 Redundancy analysis (RDA) ordination diagram (biplot) of top 20 viral populations (black) and environmental variables (blue). RDA1 explains 9.2% of variance, while RDA2 explains 6.5% of variance. Labels of data points below 0.15 have been omitted for clarity. The angles between populations and environmental factors denote their degree of correlation. Download FIG S4, JPG file, 0.2 MB. Copyright © 2021 Sun et al. 2021 Sun et al. https://creativecommons.org/licenses/by/4.0/ This content is distributed under the terms of the Creative Commons Attribution 4.0 International license.

### Host prediction.

Putative hosts were able to be predicted for 102 viral populations based on shared CRISPR spacers (Table S5). The relative abundances of these viral populations are low, all ranking below the top 3,000, and their predicted hosts also tend to be prokaryotes of low abundance.

### Read-based viral taxonomy of DEV.

**FIG 5:**
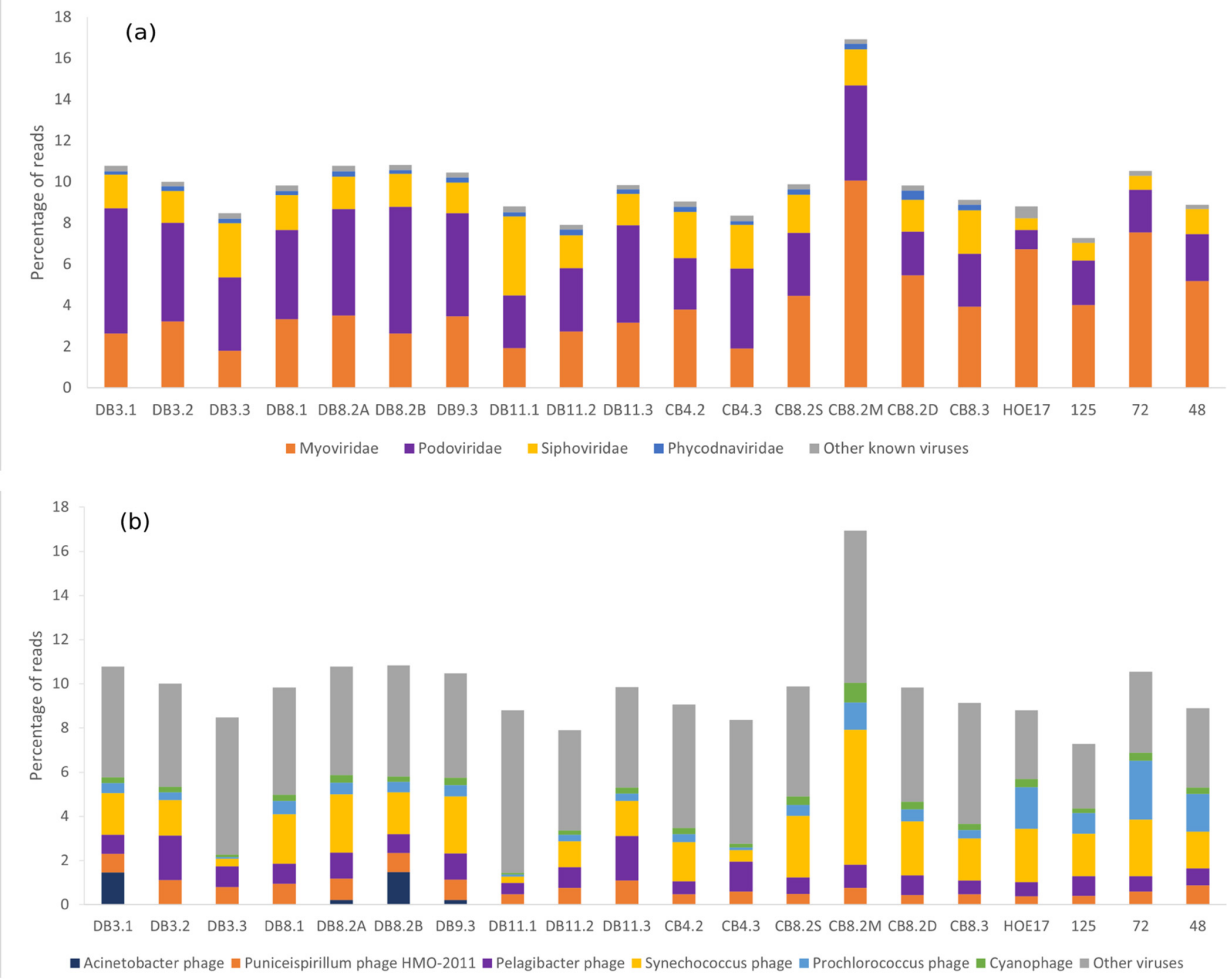
Categorization of known viruses by Kaiju read classification. (a) Relative abundance of main viral families. (b) Relative abundance of viral species categorized by presumed host. “Cyanophage” may include *Prochlorococcus* and *Synechococcus* phages. Groups of viral species assigned a certain host with low abundance (<0.05%) were omitted. The last four samples are oceanic; sample information can be found in Fig. S1 and Table S2 in the supplemental material.

Since the majority of sequences are unable to be connected to known viral taxa, separate analyses were conducted for reads assigned to known viruses and viral contigs in general. Kaiju assigned ca. 10% of trimmed reads to known viruses in all the DEV samples except for CB8.2M (Fig. 5a and b). The proportion of reads matching representative viral groups (Acinetobacter phage, *Puniceispirillum* phage, *Pelagibacter* phage, *Synechococcus* phage, *Prochlorococcus* phage, unknown cyanophage) is markedly lower in samples DB3.3 and DB11.1 (Fig. 5b). Viruses infecting other hosts were omitted from Fig. 5b due to low abundance (<0.05%).

10.1128/mSystems.01020-20.1 FIG S1 Map of oceanic samples used in viral taxonomy analysis. The map was created using Ocean Data View (R. Schlitzer, https://odv.awi.de, 2019). Download FIG S1, TIF file, 0.9 MB. Copyright © 2021 Sun et al. 2021 Sun et al. https://creativecommons.org/licenses/by/4.0/ This content is distributed under the terms of the Creative Commons Attribution 4.0 International license.

At the family level, the majority of reads were assigned to the order *Caudovirales*, with a lower proportion of *Siphoviridae* than of the other two families (Fig. 5a). Viral taxonomy at the family level is fairly stable across different samples, although the Chesapeake Bay appears to have a higher relative abundance of *Myoviridae* than the Delaware Bay, with sample CB8.2M showing an especially high proportion of myoviruses and DB11.1 showing a relatively higher proportion of *Siphoviridae* (Fig. 5a).

**FIG 6:**
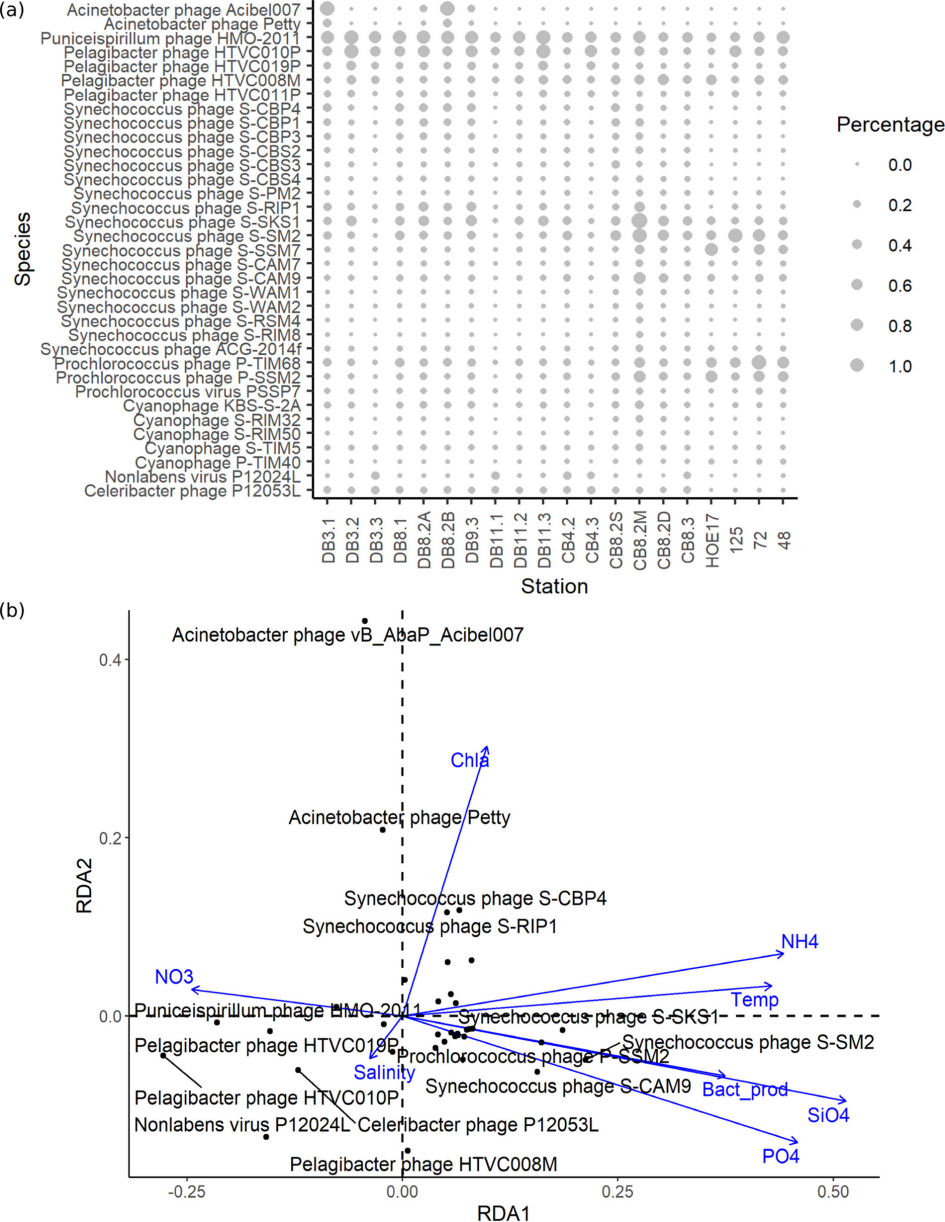
Taxonomy of known viruses by Kaiju read classification. (a) Bubble plot of most abundant viral species (greater than 0.1% reads) in DEV. Sizes of bubbles correspond to the percentages of reads that are binned to the virus species. The last four samples are oceanic; sample information can be found in Fig. S1 and Table S2 in the supplemental material. (b) Redundancy analysis (RDA) ordination diagram (biplot) of abundant viral species (black) in DEV and environmental variables (blue). RDA1 explains 33% of variance, while RDA2 explains 28% of variance. Labels of data points below 0.1 have been omitted for clarity. The angles between virus species and environmental factors denote their degree of correlation.

When the viruses were categorized by the host they are presumed to infect, cyanophages were found to be prevalent in the estuaries and more abundant during warmer seasons (Fig. 5b and 6a). The CB8.2M sample shows a large number of *Synechococcus* phages (Fig. 5b). The most abundant cyanophages in the DEV tend to be related to those isolated from the North Atlantic Ocean or the Chesapeake Bay (Fig. 6a). A small fraction (<1%) of *Prochlorococcus* phage sequences were present in almost all estuarine samples (Fig. 5b). *Pelagibacter* phage and *Puniceispirillum* phage comprise a large proportion of reads (up to 3%) (Fig. 5b) but do not show strong variation patterns throughout different samples, despite strong salinity gradients (Table 2; Fig. 6a).

Redundancy analysis (RDA) indicated the degree of correlation between abundant viral species and environmental factors. As expected, viruses are generally grouped according to their putative hosts, with all cyanophages, pelagiphages, and Acinetobacter phages clustered near each other on the biplot (Fig. 6b). Acinetobacter phages are outliers compared to other abundant species in terms of their relationship with environmental variables and are positively correlated with chlorophyll *a* concentration. *Pelagibacter* phages and *Puniceispirillum* phages exhibited a positive correlation with salinity, while cyanophages presented a positive correlation with temperature, NH_4_^+^, SiO_4_^−^, and PO_4_^3−^ concentrations and a negative correlation with NO_3_^−^ concentrations (Fig. 6b).

### Viral taxonomy of estuarine viromes versus open ocean viromes.

The percentages of known viruses (ca. 10%) were similar between the DEV samples and the four ocean samples (Fig. 5a and b). On the family level, a higher proportion of *Myoviridae* were found in oceanic samples; *Phycodnaviridae* were found in all estuarine samples but were not detected in oceanic samples (Fig. 5a). Oceanic samples contained significantly more *Prochlorococcus* phage than the estuarine environments (Fig. 5a). *Puniceispirillum* phage and *Pelagibacter* phage appear to more abundant in the estuarine environment than in open oceans (Fig. 5b). Despite differences in sampling methods across different cruises, the viral taxonomy results were comparable due to the similar sequencing technologies employed, lending reasonable legitimacy to the viral taxonomy methods used in this study.

## DISCUSSION

### The Delmarva Estuarine Virome (DEV).

Our study revealed the diversity of the double-stranded DNA (dsDNA) virioplankton communities in the Delaware Bay and Chesapeake Bay using high-throughput sequencing. Previously, the virioplankton community structure in the Chesapeake Bay was studied by sequence analysis of one sample pooled from 9 different locations of the bay, which was the first metagenomics attempt to study the estuarine virioplankton (10). However, the metagenomic sample was sequenced using Sanger technology, and thus it could not provide sufficient sequencing coverage for an in-depth assessment of the viral community structure.

Compared to other recent marine viral metagenomic data sets, the DEV returned similar sequencing quality and its sequence processing methods are up to date, producing 288 Gb of sequencing data (see Table S6 in the supplemental material). This is the first systematic study about spatial and temporal variation of virioplankton communities in estuaries using deep high-throughput sequencing. It is also one of the highest-quality viral metagenomic data sets to date, showing remarkably consistent sequencing depth and quality across samples, allowing us to discover the patterns described above.

10.1128/mSystems.01020-20.10 TABLE S6 Comparison of recent marine viral metagenomic data sets. Abbreviations: POV, Pacific Ocean Virome; TOV, Tara Ocean Virome (16–18); TOPC, Tara Oceans Polar Circle; GOV, Global Ocean Virome; DEV, Delmarva Estuarine Virome. GOV 2.0 consists of TOV, Malaspina, and TOPC (19). Download Table S6, DOCX file, 0.01 MB. Copyright © 2021 Sun et al. 2021 Sun et al. https://creativecommons.org/licenses/by/4.0/ This content is distributed under the terms of the Creative Commons Attribution 4.0 International license.

### Known and unknown viruses in the DEV.

Due to the large proportion of unknown viruses in metagenomic data sets, the analyses of known viruses and abundant viruses were handled separately. In accordance with other viral metagenomic studies, the majority of trimmed reads remain unclassified; only 10% of reads were assigned to viruses, while this value for other viromes ranges from 0.74% to 21% (21, 22). Approximately 26% of reads were mapped to viral populations (Table S3), indicating that the viral populations encompass significantly more of the sequence data than known RefSeq viruses. This proportion echoes a global viromic study where only 25% of predicted proteins were found to have similarity with any known viral proteins (20), suggesting that the majority of viral sequences are still unknown.

Compared to the dramatically changing unknown viral populations, the composition of the known viral community is relatively more stable throughout different seasons and locations in the estuaries (Fig. 3 and 6a). Attempts to identify the most abundant viral populations in the DEV found them to be mostly novel and unable to be matched to cultured viral isolates (Table 3). This implies that the most dynamic and abundant viral species in the estuaries have not yet been characterized. Indeed, the failure of known CRISPR spacers to predict hosts of abundant viral populations (total FPKM, >100) further indicates the novelty of the most prolific species in the DEV (Table S5). The spatiotemporal pattern of these abundant but uncultivated viruses is more variable than that of cultured viruses.

10.1128/mSystems.01020-20.9 TABLE S5 Predicted hosts using CRISPR. Total FPKM is the FPKM of all 16 samples added together. Download Table S5, XLSX file, 0.02 MB. Copyright © 2021 Sun et al. 2021 Sun et al. https://creativecommons.org/licenses/by/4.0/ This content is distributed under the terms of the Creative Commons Attribution 4.0 International license.

### Spatiotemporal pattern of estuarine virioplankton.

The relative abundance of viral populations varied greatly throughout different seasons in the Delaware Bay (Fig. 3), supporting the “seed bank model” which states that most viruses exist in an inactive status throughout the year while only the most abundant viruses are active in a given community (40). It has been found that about half of the Delaware Bay bacterial community cycles between rare and abundant species, with rare bacteria acting as a “seed bank” waiting for conditions to change (41). Our results showed that the Delaware Bay viral community displays a pattern similar to that of its bacterial community, which is also consistent with a previous viromics study (42).

It was difficult to discern a variation pattern in the Chesapeake Bay due to the low number of samples and the lack of upper bay sites. CB8.2M showed a significantly higher proportion of known viral reads than other samples (Fig. 5a and b) but did not show high amounts of reads mapping to the most abundant viruses (Fig. 3), further indicating that known viruses follow different patterns than abundant viruses.

In general, the bacterioplankton community in the Delaware Bay varies drastically along the salinity gradient, the dominant bacteria changing from *Actinobacteria* and *Verrucomicrobia* in the upper estuary to *Pelagibacter* and *Rhodobacterales* in the lower estuary, the community showing a clear shift from a “freshwater” profile to an “oceanic” profile (43). In contrast, although also variable, the virioplankton community does not show such a distinct transition from upper to lower estuary (Fig. 3, 5b, and 6a). This is supported by the finding that location in the estuary is not a significant factor in community similarity (Fig. 4). This is perplexing, given that viruses are dependent on their hosts for replication, but our identification of viruses may be skewed since freshwater viruses are poorly characterized in comparison to marine viruses, while bacteria in both environments are better characterized in general (44).

Despite the geographic proximity of the two estuaries, the viral community in the Delaware Bay is significantly different from that in the Chesapeake Bay (Fig. 4). The viral population difference between the two bays is more distinct than the viral population difference caused by similar temperature or salinity (Fig. 4b). This distinction may be a result of the various abiotic differences between the two estuaries, including the larger watershed and nutrient limitation in the Chesapeake Bay (33). In the Delaware Bay, abundance patterns of both known and unknown viruses appear to be variable along the salinity gradient in the spring and fall but relatively consistent from the upper to lower bay in the summer (Fig. 3 and 6a). This spatial and seasonal pattern is more pronounced in the unknown viruses, which display more dramatic changes (Fig. 3). The primary source of freshwater in the Delaware Bay is the Delaware River, and high levels of river discharge during the spring cause stratification in the estuary, impacting the spatial variation of phytoplankton production and leading to variation in the microbial community along the salinity gradient (45). On the contrary, in the summer, lower levels of discharge allow for better mixing and more consistent phytoplankton production levels along the Delaware estuary, leading to a more stable microbial community. In contrast to the Delaware Bay, such spatial and seasonal abundance patterns are obscured for the partially mixed Chesapeake Bay due to the number of tributaries along its length and its relatively long water residence time (∼180 days) (30). An interannual study found that viral abundance and viral production did not change greatly from the upper to the lower Chesapeake Bay, despite strong environmental gradients (46). The DEV relative abundance data concur by showing little influence from salinity gradients in the Chesapeake Bay, although this may be due to the lack of upper bay samples in this study (Fig. 3). The inclusion of different sampling depths in the Chesapeake Bay but not the Delaware Bay is also a contributor to the statistical dissimilarity between the viral populations of the two bays (Table 2; Fig. 4). The spatiotemporal gradients have allowed us to reveal the above-described patterns in the estuarine virome.

In several of the analyses conducted in this study, samples DB3.3 and DB11.1 showed a similar community structure that is distinct from that of the other DEV samples. A lower percentage of known viruses was identified in these two samples (Fig. 5a and b), and correspondingly higher abundances of unknown viruses were observed (Fig. 3). These two samples were grouped together and away from the other samples, both in the qualitative cluster network plot of viral contigs (Fig. 2) and the nonmetric multidimensional scaling (NMDS) plot of abundant viral populations (Fig. 4a). Analysis of variance (ANOSIM) testing showed significant dissimilarity when these two samples were grouped together versus other samples (Fig. 4b). The different community structures of these two samples may be indicative of some episodic event in the Delaware Bay, the cause of which is not documented in the environmental factors to which we currently have access (see ”Data availability“ in Materials and Methods).

### Comparison of the DEV with other estuarine and oceanic viromes.

The abundance of viruses in the sea is around 15-fold higher than that of bacteria and archaea, which matches our observations (Fig. 1) (47). Other studies also found viral counts and cell counts to be positively correlated to temperature in the Chesapeake Bay and observed stronger seasonal variation than spatial variation (46).

**FIG 7:**
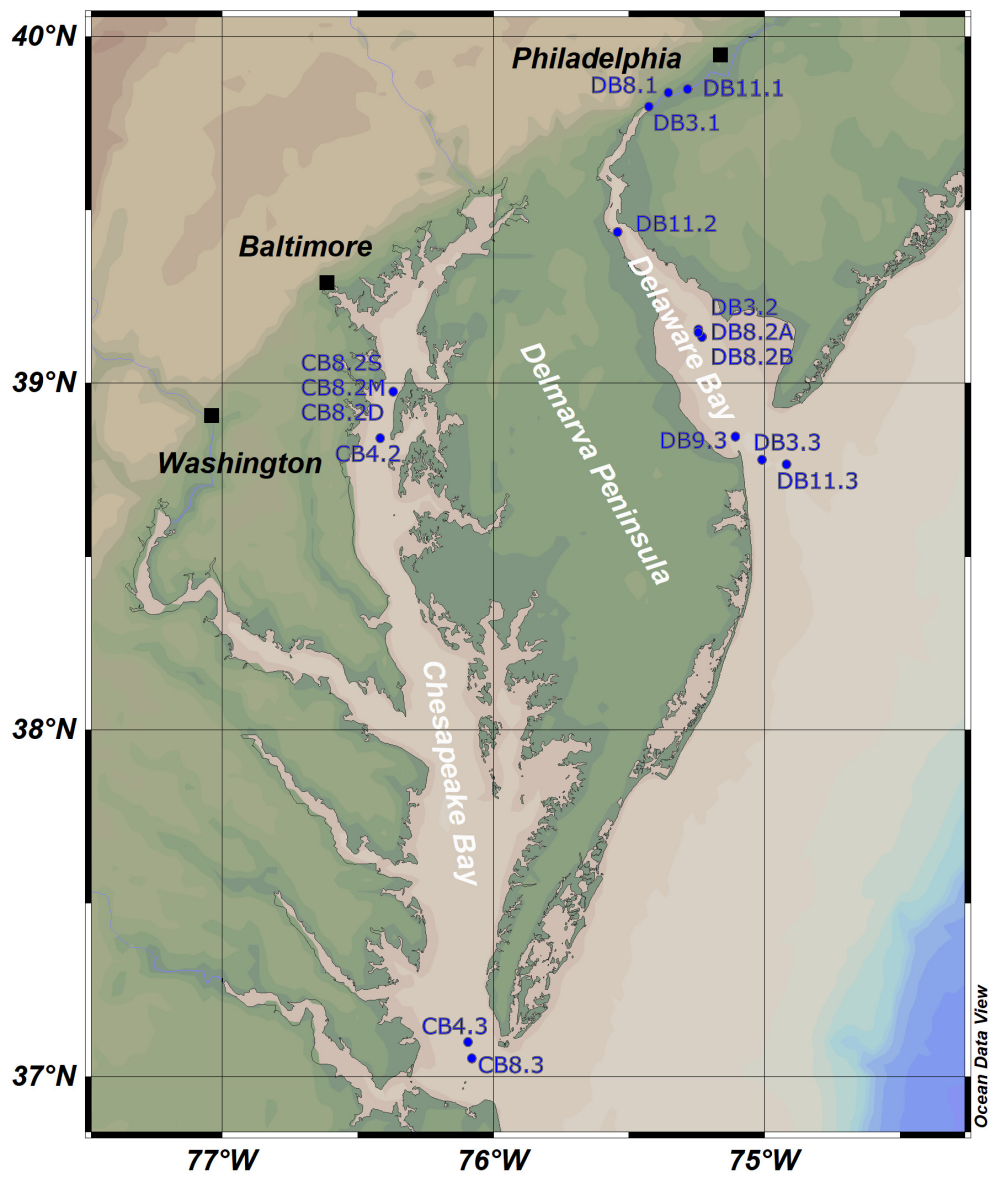
Sampling map for Delmarva Estuarine Virome (DEV) on the East Coast of North America. The map was created using Ocean Data View (R. Schlitzer, https://odv.awi.de, 2019), with the ETOPO1 map (87).

On the family level, members of the viral family *Myoviridae* are generally found to be most abundant in the open ocean, followed by those from the *Podoviridae*, while *Siphoviridae* family viruses are less common (48). Estuaries appear to follow an overall similar trend. The higher proportion of *Siphoviridae* in DB11.1 may be influenced by terrestrial runoff at its high, riverine position (Fig. 5a and see Fig. 7). Estuarine samples from the Global Ocean Sampling (GOS) viral metagenomic study found that the Chesapeake Bay has a higher relative abundance of *Myoviridae* than the Delaware Bay (15), which concurs with our results (Fig. 5a). Since then, a viral community study involving both the Delaware Bay and the Chesapeake Bay has not been conducted. An early study of the Chesapeake Bay found that the proportion of *Siphoviridae* is much lower than that of *Myoviridae* and *Podoviridae* and that viruses with eukaryotic hosts rarely occur (10), which is consistent with this study (Fig. 5a). Other estuarine viromes in Korea and the Baltic Sea also showed high proportions of *Myoviridae* and *Podoviridae* members (22, 25, 49), although a study in China found higher proportions of *Siphoviridae* than *Myoviridae* in an estuary (21). This shows that virioplankton in estuaries around the world have a similar structure on the family level. In this study, a higher proportion of *Myoviridae* was found in oceanic samples than in estuarine samples; the relatively higher proportion of *Myoviridae* in CB8.2M and CB8.2D may be due to the influence of oceanic water from vertical stratification, as is evidenced by their higher salinity than that of the surface water sample (Fig. 5a; Table S1). Cyanomyoviruses are more abundant than cyanopodoviruses in coastal and open ocean viral metagenomes than in viral metagenomes in estuaries (50). Since a large portion of known viruses in the DEV are cyanophages (Fig. 5b), this supports our current findings. *Phycodnaviridae* are abundant and ubiquitous in the oceans, but this study did not find *Phycodnaviridae* in oceanic sites (51). The absence of *Phycodnaviridae* in oceanic sites in this study may be due to differing bioinformatic methods used. Since members of the *Phycodnaviridae* are larger than those of the *Caudovirales*, with capsid sizes ranging from 100 to 220 nm (52), it may also be due to the difference in viral sampling techniques on different cruises.

Cyanophages and pelagiphages are thought to be the most abundant known viruses in marine environments (53). The higher prevalence of cyanophage in the summer and large proportions of *Pelagibacter* phage and *Puniceispirillum* phage are consistent with other estuarine viromic studies (Fig. 5b) (21, 22). *Pelagibacter* comprises 40 to 60% of the bacterioplankton community in mid- to lower Delaware Bay and is significantly less abundant in the upper bay, comprising 0 to 5% of metagenomic reads (B. Campbell, unpublished data); meanwhile, pelagiphage make up only 1 to 2% of total reads and about 10% of known viral reads and do not show a clear transition from the upper to the lower bay, displaying completely different patterns than their presumed hosts (Fig. 5b). Since isolation of pelagiphage is difficult and sometimes requires methods such as single-cell genomics (54, 55), our current ability to identify pelagiphages from metagenomic sequences is highly limited and may be causing this discrepancy between phage and host. Cyanophages play an important role in the regulation of cyanobacterial abundance in the Chesapeake Bay (56). The most abundant cyanophage species in DEV matched some *Synechococcus* phages isolated from the Chesapeake Bay, including the podoviruses *Synechococcus* phage S-CBP1, S-CBP3, and S-CBP4 and the siphoviruses *Synechococcus* phage S-CBS2, S-CBS3, and S-CBS4 (57) (Fig. 6a). All of these cyanophages are highly host specific, infecting locally isolated *Synechococcus* species CB0101, CB0204, and CB0202 (57). Unlike for pelagiphage, the extensive cyanophage isolation work conducted in the geographic vicinity allows us to make more connections between phage and host. We anticipate similar findings for *Pelagibacter* phage-host relationships with the isolation and documentation of more pelagiphage strains. In contrast with the broad distribution of *Synechococcus*, *Prochlorococcus* is rarely found in coastal eutrophic systems but is abundant in warm oligotrophic waters (58). The significant presence of *Prochlorococcus* phage in oceanic samples compared to estuarine samples (Fig. 5b) supports this paradigm and is consistent with previous studies (50). The small fraction (<1%) of *Prochlorococcus* phage sequences found in estuarine samples (Fig. 5b) may be due to the fact that certain cyanophages such as cyanomyoviruses tend to cross-infect *Synechococcus* and *Prochlorococcus* (59). The host ranges of current phage isolates were explored to differing degrees, so a cyanophage isolated using *Prochlorococcus* does not indicate that it does not also infect *Synechococcus*.

The most abundant viral populations in the DEV tend to be very novel, which concurs with other contig-level virome studies (20, 48). Abundant marine viral populations have been found to be both variable and persistent across seasons (48) and locations (16, 18). Similarly, abundant viral populations in the DEV were found to have various patterns across samples (Fig. 3). Despite most of these populations being unknown, their dominance in the estuarine environment suggests that they may infect some abundant bacterial populations which have not yet been identified. Since unknown viral populations account for a large portion of these estuarine viromes, and their potential hosts and ecological role still remain largely unknown, it is necessary to understand more about these cryptic viral groups.

### Importance of single-cell and single-molecule methods.

Phages infecting abundant but relatively slow-growing and difficult-to-culture marine bacteria make up a significant portion of marine viruses in the ocean (60). Since 2017, uncultivated virus genomes have outnumbered virus genomes sequenced from isolates (61), but identification of metagenomic sequences still relies primarily on culture-dependent microbial discovery. In recent years, single-cell genomics have offered valuable insights into the marine viral community (62), discovering some of the most abundant and ecologically significant viruses in the marine ecosystem (37, 38). In particular, the abundance of the single virus isolate 37-F6, of which the putative host is *Pelagibacter* (55), is thought to rival or exceed that of *Pelagibacter* phage HTVC010P and *Puniceispirillum* phage HMO-2011, which were previously thought to be the most abundant viruses in the ocean (38, 54, 63). Likewise, long-read single-molecule sequencing uses long nanopore reads (20 to 80 kb) to capture entire viral genomes without assembling, avoiding some of the biases induced by short-read *de novo* assembly, thus revealing “hidden” viral diversity not covered by conventional metagenomic sequencing methods (39). Several of the most abundant viral populations in the DEV have the closest match to prokaryotes discovered using nonconventional methods such as single-cell genomics, single-virus genomics, and long-read single-molecule sequencing (Fig. 3; Table 3), demonstrating the importance of non-cultivation-dependent virus characterization methods for revealing viral diversity. These results indicate that discoveries using the above-described methods may be important for revealing the most abundant and ecologically relevant viral species in the marine and estuarine environment, improving our understanding of viral dark matter.

### Discovery of putative A. baumannii phage.

A highly abundant viral population was found in the Delaware Bay, and it had the closest match to Acinetobacter baumannii phages. Nicknamed “Iraqibacter” due to its origin in military hospitals in Iraq, A. baumannii is a multidrug-resistant pathogen that is a problem in hospitals around the world, although its natural habitat remains unknown (64–66). The clinical concern of antibiotic-resistant A. baumannii is driving phage isolation in hope of discovering potential viral strains for phage therapy, since antibiotic-resistant A. baumannii was found to be more susceptible to phage infection (67–69). As of 2018, 42 Acinetobacter phages have been isolated, and over half of their encoded proteins are of unknown function (70). Since the information is derived from a MAG (metagenome-assembled genome), it is possible that the genome may be misassembled or inaccurately annotated due to its being a novel virus (61). Nevertheless, the discovery of a putative A. baumannii phage and the fact that it appears to be exclusive to Delaware Bay suggests an episodic contamination event of hospital origin in the Delaware Bay, likely stemming from the highly polluted Delaware River. Further work is needed to characterize and explore the distribution of this novel viral population.

### Conclusions.

We were surprised to find that the virioplankton community does not show a distinct transition from upper to lower estuary or across different seasons despite strong environmental gradients, unlike their prokaryotic hosts. In contrast, Delaware Bay and Chesapeake Bay viral populations were found to be significantly different from each other, despite their geographical proximity. We found that the most abundant viral populations in estuaries (top 20) are not the usually dominant viral groups such as pelagiphage and cyanophage but are viruses which have not yet been cultivated, related to uncultured viral sequences discovered via single-cell and assembly-free long-read single-molecule methods, highlighting the importance of these unconventional methods for viral discovery. A viral contig similar to phages infecting Acinetobacter baumannii (“Iraqibacter”) was found to be highly abundant in the Delaware Bay but was not found in any other marine or estuarine environment. Comparison with other aquatic environments showed that estuarine virioplankton around the world have a similar structure on the family level (*Siphoviridae*, *Myoviridae*, *Podoviridae*), while open ocean virioplankton have a higher proportion of *Myoviridae* and *Prochlorococcus* phage. We anticipate that the further isolation of novel viral species will enhance our understanding of the estuarine virome.

## MATERIALS AND METHODS

### Sample collection and preparation.

Ten water samples were collected from the Delaware Bay in March, August/September, and November 2014, and six samples were collected from the Chesapeake Bay in April and August 2015, on board the RV *Hugh R Sharp*. Samples were collected to reflect different salinity gradients in each estuarine ecosystem (Fig. 7). The overall sampling strategy was to collect viral communities across a wide spatial and temporal scale in both estuaries. Additional information about environmental conditions can be found in Table 2 and in Table S1 in the supplemental material. Samples DB8.2A and DB8.2B are diel samples; samples CB8.2S, CB8.2M, and CB8.2D were taken at different depths (∼1, 13, and 22 m, respectively).

At each of the sampling sites, water samples were collected using a Niskin bottle on a Sealogger conductivity-temperature-depth rosette water sampler. For each sample, 10 liters of seawater was prefiltered through 0.2-μm-pore-size membrane filters (Millipore Corporation, Billerica, MA) to remove bacteria and larger organisms. Viral communities were concentrated from the 0.2-μm filtrates by following the FeCl_3_ flocculation method described by John et al. (71). Viral dsDNA was extracted using the phenol-chloroform/isoamyl method (72).

### Viral and cellular counts.

For viral and bacterial counts, 2 ml seawater was fixed at a final concentration of 0.5% glutaraldehyde at 4°C for 20 min and then stored at 4°C. Viral and bacterial abundances were determined using an Epics Altra II flow cytometer (Beckman Coulter, Miami, FL, USA) as described by Brussaard (73). The fixed samples were stained with SYBR green I (Invitrogen, CA, USA) and enumerated at event rates of 50 to 200 particles/s (bacteria) or 100 to 300 particles/s (viruses). For every sample, 10 μl of 1 mm-diameter fluorescent microspheres (Molecular Probes, Inc., OR, USA) was added as reference beads. Each sample was run twice on the flow cytometer, and the average of count values was taken. The data were analyzed by EXPOTM_32 MultiCOMP software (Beckman Coulter, Miami, FL, USA).

### DNA sequencing and metagenome assembly.

Viral DNA was sequenced using an Illumina HiSeq 2500 (Illumina, San Diego, CA, USA) at the Joint Genome Institute, U.S. Department of Energy, generating paired-end (PE) reads with a read length of 150 bp. The resulting virome collection is referred to as the Delmarva Estuarine Virome (DEV). Known Illumina adapters were removed from sequencing reads and low-quality reads (Phred quality score < 12, containing more than 3 “N’s,” or length under 51 bp) were trimmed with BBDuk (74). The remaining reads were mapped to a masked version of human HG19 with BBMap, with all hits over 93% identity discarded (74). Trimmed Illumina reads were *de novo* assembled with Megahit using a range of K-mers (75).

### Viral contig identification and annotation.

Contigs that are likely to be of viral origin were selected using the method described by Paez-Espino et al. (34). Briefly, contigs smaller than 5 kb were discarded, and ORFs were predicted for the remaining contigs and filtered based on the number of genes that they shared with those encoding known viral proteins. The resulting list of contigs was considered to be viral and was uploaded to MG-RAST and annotated using the RefSeq database (76). Rarefaction curves were generated by MG-RAST using data from the M5NR database and visualized using ggplot2 in R (76, 77).

### Viral contig cluster network.

Viral contigs were clustered with BLASTN (E value, 1 × 10^−50^; ≥90% identity; ≥75% covered length) using single linkage clustering (34). Contigs not belonging to a cluster were deemed singletons. The clusters and their interaction with the samples with which they were associated were visualized using the “prefuse force directed layout” in Cytoscape (78). Singletons were omitted from the cluster visualization for clarity.

### Viral populations and detection of circular viral contigs.

To reduce redundancy for read mapping analysis, for each viral cluster, the longest sequence within the cluster was deemed the seed sequence and was combined with the singletons to form a nonredundant viral population database. Circular viral contigs were detected using VRCA (viral and circular content from metagenomes), which finds circular contigs in metagenome assemblies by identifying read overlaps at the start/end of contigs (79). To examine chosen circular contigs of interest, a complete viral genome was reverse complemented, annotated using RAST, and visualized using DNAplotter from Artemis (36, 80).

### Relative abundance of viral populations and relationship with environmental variables.

Quality trimmed DNA reads were mapped to the nonredundant viral populations using BBMap with the mapping parameters as recommended in viromic benchmarking studies (>90% identity, >75% contig length) (81, 82). Reads were counted and normalized to FPKM (fragments per kilobase million) using SAMtools (83). FPKM is used as a proxy for relative abundance (82). Total FPKM of each sample was added together for each viral population and ranked to find the most abundant viral populations.

To explore the similarity of samples based on viral population profiles, a nonmetric multidimensional scaling (NMDS) based on Bray-Curtis dissimilarity matrices was plotted using the vegan package in R and visualized using ggplot2 (77, 84). Due to computing constraints, only the most abundant 5,000 (out of 26,487) viral populations were used for this analysis. To further quantify the similarity of viral population profiles across different groups of samples, an analysis of variance (ANOSIM) test was performed with the same 5,000 viral populations using the vegan package in R (84).

The top 20 most abundant viral populations were chosen to represent the dominant viruses in the estuaries, and their abundance was plotted using ggplot2 in R (77). To identify the top 20 viral populations, they were searched against the NCBI-nr database with BLASTN (85). To further explain the relationship between the abundance of dominant viruses and environmental variables, redundancy analysis (RDA) results were plotted for the top 20 viruses using the vegan package in R and visualized using type I scaling in ggplot2 (77, 84).

### Host prediction.

Putative hosts were predicted *in silico* by comparison of viral populations to known CRISPR (clustered regularly interspaced short palindromic repeat) spacers. The collection of CRISPR spacers from the Microbial Isolate Genomes from the IMG/M database was used as a blastn query against all of the viral populations, and hits were used if they were 100% length, allowing a maximum of 1 mismatch (85). The resulting virus-host pairings were sorted according to the total relative abundance (FPKM) of the viral populations. Quantitative analysis of cooccurrence of viral and prokaryotic communities, although potentially insightful, is beyond the scope of this paper.

### Viral taxonomy of DEV reads and relationship with environmental variables.

The analysis of known viral taxonomy was handled separately from that of abundant viral populations, in order to get a comprehensive picture of both the classified viruses and the viral “dark matter” in the estuaries. To acquire the taxonomy of known viruses, trimmed reads were classified using Kaiju (86), and taxonomy was assigned via comparison with Kaiju’s built-in “viruses” database (as of June 2019), using the default greedy mode parameters. A classification summary was created using the kaiju2table program, and percentages of reads for each taxon were used as a proxy for species relative abundance. The abundances of species with a percentage greater than 0.1% in DEV were plotted using ggplot2 in R (77). These species were categorized according to the host they are presumed to infect, derived from the species name, and may not reflect their ability to infect other potential hosts. The category “Cyanophage” may include *Prochlorococcus* and *Synechococcus* phages. All species were categorized according to family, and the top four most abundant viral families were plotted.

To explain the relationship between abundant species and environmental variables, RDA was plotted for species in DEV with a percentage greater than 0.1% in DEV using the vegan package in R and visualized using type I scaling in ggplot2 (77, 84).

### Comparison of viral taxonomy with oceanic samples.

To compare the viral compositions of estuarine and open ocean waters, the metagenomic reads of four publicly available oceanic surface water samples were downloaded and assigned taxonomy with Kaiju, using the above-described methods (19, 48). The viral metagenomic samples (from TARA Oceans, Hawaii Ocean Experiment) were chosen due to their similar sequencing technology and depth and their wide global distribution (Fig. S1; Table S2).

10.1128/mSystems.01020-20.6 TABLE S2 Sampling conditions of oceanic samples used in viral taxonomy analysis. Download Table S2, DOCX file, 0.01 MB. Copyright © 2021 Sun et al. 2021 Sun et al. https://creativecommons.org/licenses/by/4.0/ This content is distributed under the terms of the Creative Commons Attribution 4.0 International license.

### Data availability.

Environmental conditions can be found at http://dmoserv3.bco-dmo.org/jg/serv/BCO-DMO/Coast_Bact_Growth/newACT_cruises_rs.html0%7Bdir=dmoserv3.whoi.edu/jg/dir/BCO-DMO/Coast_Bact_Growth/,info=dmoserv3.bco-dmo.org/jg/info/BCO-DMO/Coast_Bact_Growth/new_ACT_cruises%7D.

The metagenomic sequences are available in the IMG database (https://img.jgi.doe.gov/) under the study name “Aqueous microbial communities from the Delaware River/Bay and Chesapeake Bay under freshwater to marine salinity gradient to study organic matter cycling in a time-series” (GOLD Study ID Gs0114433; GOLD project IDs Gp0112820 to Gp0112829 for the 10 Delaware Bay samples and Gp0123713 to Gp0123718 for the 6 Chesapeake Bay samples).
