# Supplementary material for: Uncultivated Viral Populations Dominate Estuarine Viromes on the Spatiotemporal Scale
Source: mSystems. 2021 Mar 16;6(2):e01020-20. doi: 10.1128/mSystems.01020-20 (PMC8546989; doi:10.1128/mSystems.01020-20)
Supplement: TABLE S3 [file msystems.01020-20-st003.docx]

**Table S3.** Number of viral clusters and singletons, and percentage of trimmed reads that map to viral populations.

| **Sample** | **Viral contigs** | **Unique clusters** | **Singletons** | **Mapped percentage** |
| --- | --- | --- | --- | --- |
| DB3.1 | 2666 | 2065 | 439 | 27% |
| DB3.2 | 4521 | 2960 | 1353 | 32% |
| DB3.3 | 2645 | 1066 | 1472 | 24% |
| DB8.1 | 2846 | 697 | 1026 | 24% |
| DB8.2A | 3025 | 2307 | 536 | 26% |
| DB8.2B | 2650 | 2070 | 419 | 27% |
| DB9.3 | 3909 | 2535 | 1137 | 27% |
| DB11.1 | 2119 | 1046 | 1000 | 18% |
| DB11.2 | 5374 | 2106 | 3115 | 25% |
| DB11.3 | 3910 | 2776 | 900 | 32% |
| CB4.2 | 2309 | 1608 | 623 | 24% |
| CB4.3 | 1770 | 2144 | 542 | 26% |
| CB8.2S | 2661 | 1282 | 1173 | 28% |
| CB8.2M | 2842 | 823 | 1968 | 30% |
| CB8.2D | 2548 | 1102 | 1491 | 25% |
| CB8.3 | 2395 | 1647 | 651 | 24% |
