# Supplementary figures and images for: Uncultivated Viral Populations Dominate Estuarine Viromes on the Spatiotemporal Scale
Source: mSystems. 2021 Mar 16;6(2):e01020-20. doi: 10.1128/mSystems.01020-20 (PMC8546989; doi:10.1128/mSystems.01020-20)

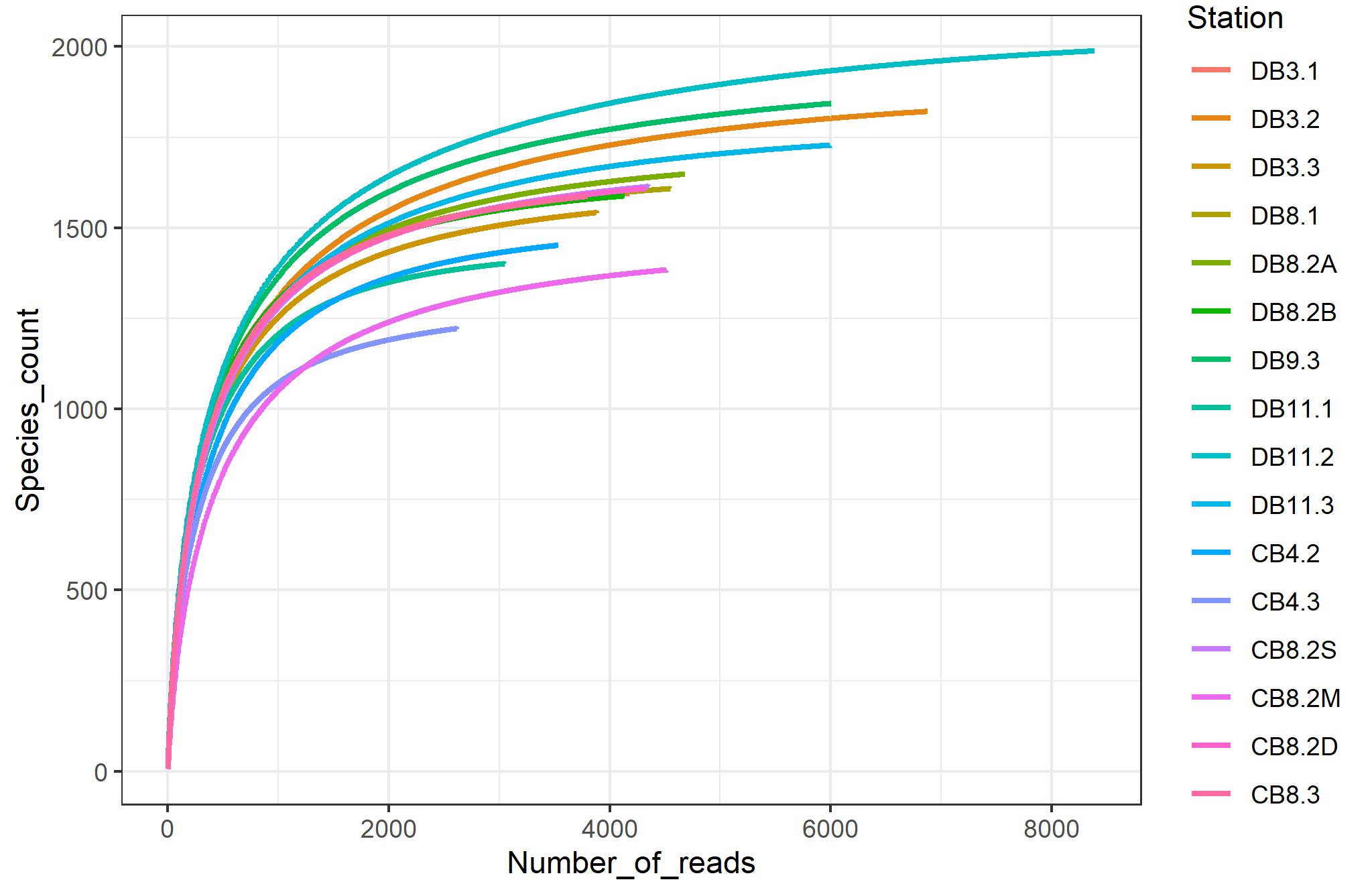

Supplement: FIG S2 [file msystems.01020-20-sf002.jpg]

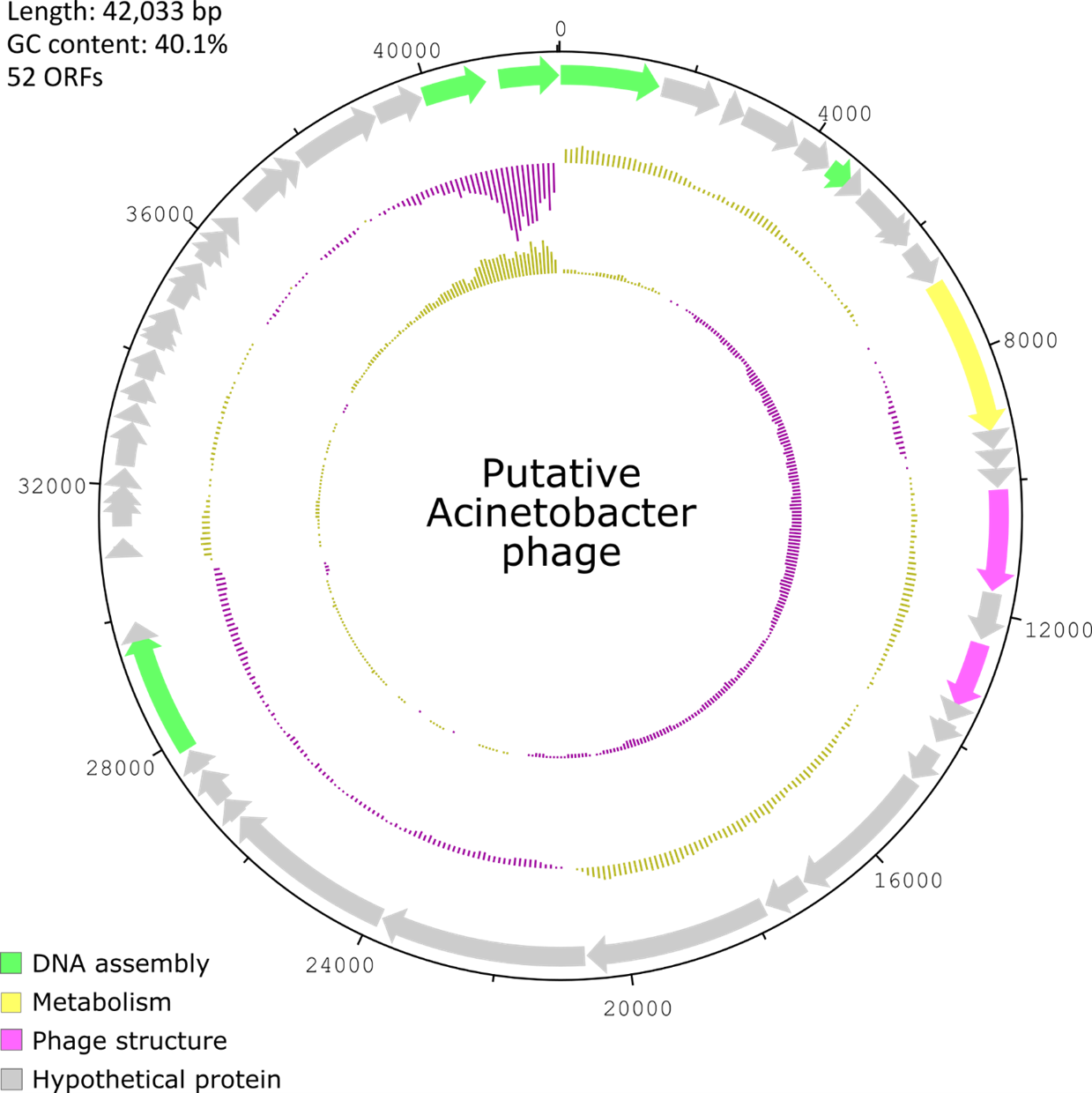

Supplement: FIG S3 [file msystems.01020-20-sf003.tif]

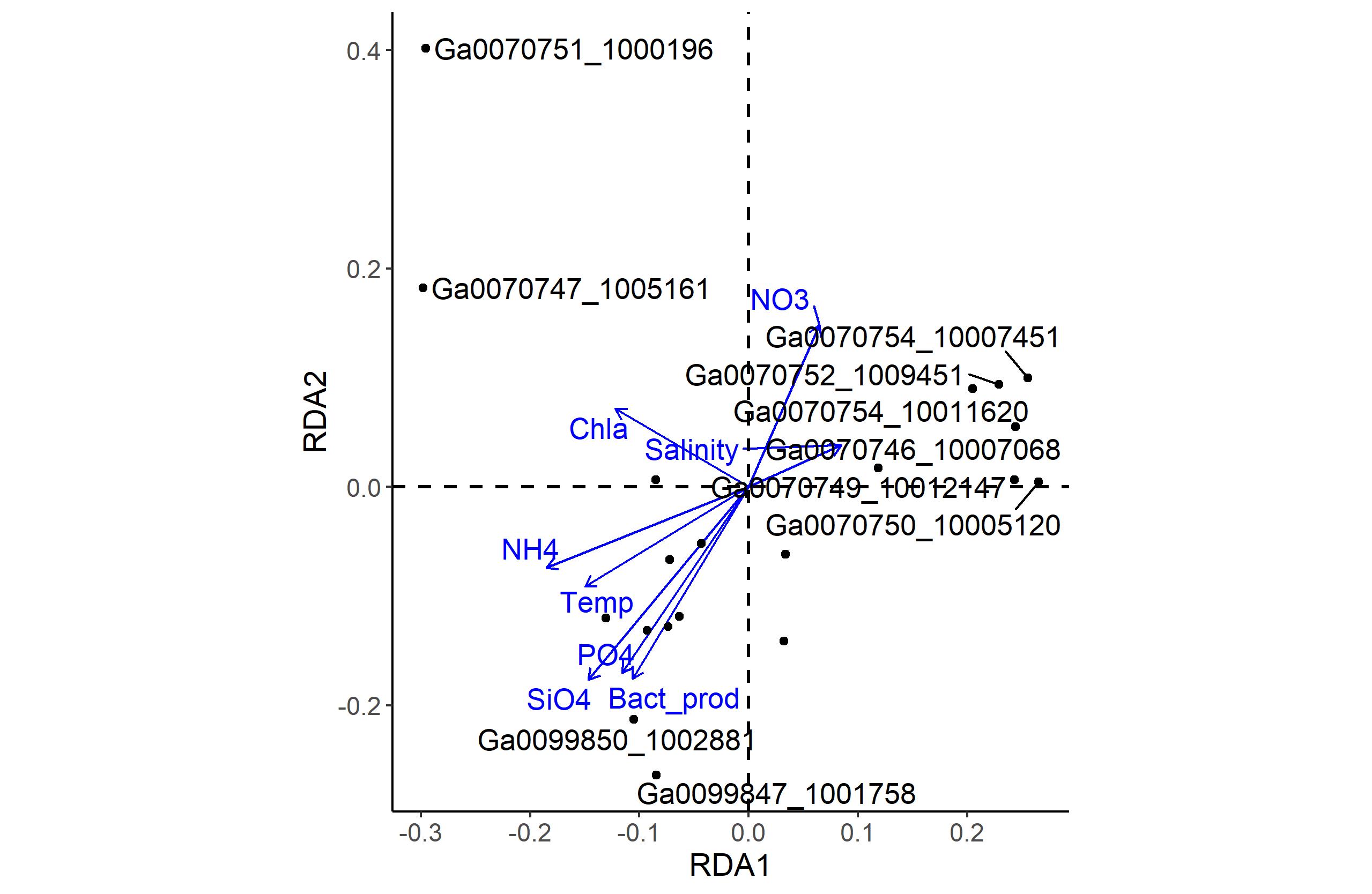

Supplement: FIG S4 [file msystems.01020-20-sf004.jpg]

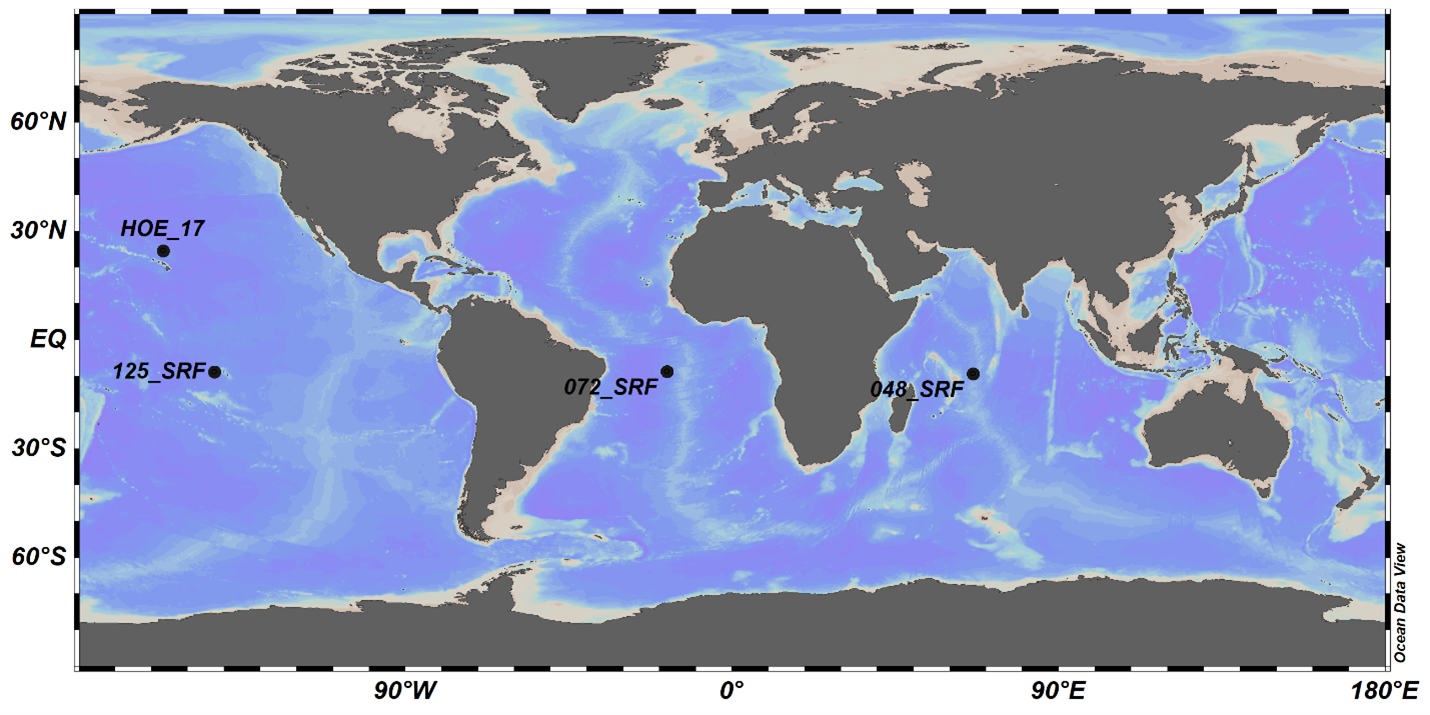

Supplement: FIG S1 [file msystems.01020-20-sf001.tif]
