# Supplementary material for: Uncultivated Viral Populations Dominate Estuarine Viromes on the Spatiotemporal Scale
Source: mSystems. 2021 Mar 16;6(2):e01020-20. doi: 10.1128/mSystems.01020-20 (PMC8546989; doi:10.1128/mSystems.01020-20)
Supplement: TABLE S4 [file msystems.01020-20-st004.docx]

**Table S4** Length distribution of viral populations.

| Total viral populations | 26,487 |
| --- | --- |
| Viral populations > 10 kb | 12,531 |
| Viral populations > 25 kb | 2,523 |
| Viral populations > 50 kb | 353 |
| Total length (bp) | 346,627,485 |
| Largest contig (bp) | 186,740 |
| N50 (bp) | 15,181 |
| N75 (bp) | 9,232 |
| L50 | 6,505 |
| L75 | 13,896 |
