# Supplementary material for: Uncultivated Viral Populations Dominate Estuarine Viromes on the Spatiotemporal Scale
Source: mSystems. 2021 Mar 16;6(2):e01020-20. doi: 10.1128/mSystems.01020-20 (PMC8546989; doi:10.1128/mSystems.01020-20)
Supplement: TABLE S6 [file msystems.01020-20-st006.docx]

**Table S6.** Comparison of recent marine viral metagenomic datasets. Abbreviations: Pacific Ocean Virome (POV); Tara Ocean Virome (TOV); Tara Oceans polar circle (TOPC); Global Ocean Virome (GOV); Delmarva Estuarine Virome (DEV). GOV 2.0 consists of TOV, Malaspina and TOPC.

|  | POV  (2013) | TOV  (2015) | Malaspina  (2016) | TOPC  (2019) | DEV  (This study) |
| --- | --- | --- | --- | --- | --- |
| # of metagenomes | 32 | 43 | 14 | 41 | 16 |
| Average # of reads per metagenome | 188,128 | 100,706,767 | 28,334,677 | 53,500,000 | 120,278,861 |
| Read length (bp) | 310 | 101 | 151 | 101 | 151 |
| Sequencing platform | 454 Titanium | Illumina Hiseq 2000 | Illumina Hiseq | Illumina Hiseq  2000 | Illumina HiSeq 2500 1TB |
| Average # of contigs per sample | NA | 88,878  (SOAP denovo) | Unknown | Unknown | 962,521  (Megahit) |
| Average # of viral contigs per sample | NA | 5852 (GOV 2.0) | | | 3012 |
