# Supplementary material for: Uncultivated Viral Populations Dominate Estuarine Viromes on the Spatiotemporal Scale
Source: mSystems. 2021 Mar 16;6(2):e01020-20. doi: 10.1128/mSystems.01020-20 (PMC8546989; doi:10.1128/mSystems.01020-20)
Supplement: TABLE S2 [file msystems.01020-20-st002.docx]

**Table S2.** Sampling Conditions of oceanic samples used in viral taxonomy analysis.

| **Dataset** | **Sample** | **Date** | **Local Time** | **Region** | **Temp (°C)** | **Salinity (ppt)** |
| --- | --- | --- | --- | --- | --- | --- |
| GOV 2.0 | 125_SRF | 2011-08-08 | 17:33 | Mid-Pacific | 27 | 35 |
| GOV 2.0 | 072_SRF | 2010-10-05 | 08:00 | Mid-Atlantic | 25 | 36 |
| GOV 2.0 | 048_SRF | 2010-04-19 | 07:56 | Indian Ocean | 30 | 34 |
| HOE | HOE_17 | 2015-07-27 | 10:05 | Pacific (Hawaii) | 27 | 35 |
